# An exploration of how developers use qualitative evidence: content analysis and critical appraisal of guidelines

**DOI:** 10.1186/s12874-020-01041-8

**Published:** 2020-06-17

**Authors:** Yun-Yun Wang, Dan-Dan Liang, Cui Lu, Yue-Xian Shi, Jing Zhang, Yue Cao, Cheng Fang, Di Huang, Ying-Hui Jin

**Affiliations:** 1 Center for Evidence-Based and Translational Medicine, Zhongnan Hospital of Wuhan University, Wuhan, China; 2 Department of Evidence-Based Medicine and Clinical Epidemiology, The Second Clinical College, Wuhan University, Wuhan, China; 3 Center for Evidence-Based and Translational Medicine, Wuhan University, Wuhan, China; 4 School of Nursing and Health, Henan University, Kaifeng, China; 5 Department of surgery, Huaihe Hospital of Henan University, Kaifeng, China; 6 Center for Evidence-based Medicine, Institute of Evidence-Based Medicine and Knowledge Translation, Henan University, Kaifeng, China; 7 Emergency Department, Tianjin TEDA hospital, Tianjin, China; 8 School of Nursing, Peking University, Beijing, China; 9 First Clinical College of Wuhan University, Wuhan, China

**Keywords:** Qualitative research, Healthcare, Guideline development, AGREE II

## Abstract

**Background:**

Clinical practice guidelines have become increasingly widely used to guide quality improvement of clinical practice. Qualitative research may be a useful way to improve the quality and implementation of guidelines. The methodology for qualitative evidence used in guidelines development is worthy of further research.

**Methods:**

A comprehensive search was made of WHO, NICE, SIGN, NGC, RNAO, PubMed, Embase, Web of Science, CNKI, Wanfang, CBM, and VIP from January 1, 2011 to February 25, 2020. Guidelines which met IOM criteria and were focused on clinical questions using qualitative research or qualitative evidence, were included. Four authors extracted significant information and entered this onto data extraction forms. The Appraisal of Guidelines for Research and Evaluation (AGREE II) tool was used to evaluate the guidelines’ quality. The data were analyzed using SPSS version 17.0 and R version 3.3.2.

**Results:**

Sixty four guidelines were identified. The overall quality of the guidelines was high (almost over 60%). Domain 1 (Scope and Purpose) was ranked the highest with a median score of 83% (IQ 78–83). Domain 2 (Stakeholder involvement) and Domain 5 (Applicability) were ranked the lowest with median scores of 67% (IQ 67–78) and 67% (IQ 63–73) respectively. 20% guidelines used qualitative research to identify clinical questions. 86% guidelines used qualitative evidence to support recommendations (mainly based on primary studies, a few on qualitative evidence synthesis). 19% guidelines applied qualitative evidence when considering facilitators and barriers to recommendations’ implementation. 52% guideline developers evaluated the quality of the primary qualitative research study using the CASP tool or NICE checklist for qualitative studies. No guidelines evaluated the quality of qualitative evidence synthesis to formulate recommendations. 17% guidelines presented the level of qualitative research using the grade criteria of evidence and recommendation in different forms such as I, III, IV, very low. 28% guidelines described the grades of the recommendations supported by qualitative and quantitative evidence. No guidelines described the grade of recommendations only supported by qualitative evidence.

**Conclusions:**

The majority of the included guidelines were high-quality. Qualitative evidence was mainly used to identify clinical questions, support recommendations, and consider facilitators and barriers to implementation of recommendations’. However, more attention needs to be paid to the methodology. For example, no experts proficient in qualitative research were involved in guideline development groups, no assessment of the quality of qualitative evidence synthesis was included and there was lack of details reported on the level of qualitative evidence or grade of recommendations.

## Background

Qualitative research can be defined as research that involves “the collection, analysis and interpretation of data that are not easily reduced to numbers; these data relate to the social world and the concepts and behaviors of people within it” [1]. Data from qualitative research can address certain types of significant questions that may not be answered by quantitative research methods, such as “how” and “why”a given intervention engenders its effects. Qualitative research is now widely used for a variety of purposes in the field of healthcare, for example, the identification of patients’ concerns, the manner in which people select and use healthcare services, and the circumstances under which healthcare interventions play a role in practice [2, 3].

Taking the merits of qualitative research into account, it has attracted the attention of guideline developers and is gradually becoming accepted to inform guideline recommendations, for example WHO (World Health Organization) has affirmed in its handbook for guideline development that qualitative evidence should be considered and used in the process of guideline development and the WHO Guidelines Review Committee (GRC) internet site also provides additional guidance on when and how to use qualitative research data to inform WHO guidelines [4]. Many professional scholars and researchers have also used qualitative research or evidence to conduct projects on the development and implementation of guidelines such as addressing questions about the values and preferences of relevant stakeholders (e.g., patients, caregivers, and the public), the acceptability and feasibility of the interventions and the influence of the interventions on equity and human rights [4–9]. This provides opportunities for qualitative research methodologists to be involved in the process of developing guideline recommendations [10, 11] and exploring facilitators of and barriers to the guideline’s implementation [12].

As Lewin & Glenton said, qualitative research may be entering a new era of being used in the process of guideline development, and it is beneficial for decision making [13]. Our aim was to further understanding of the way qualitative evidence has been used in the process of the existing guideline development process, for example, whether qualitative evidence was retrieved or how many recommendations are supported by qualitative evidence. To achieve this we conducted a systematic search, a rigorous quality evaluation of guidelines, and comprehensive information extraction related to qualitative evidence in guidelines. We also performed content analysis for the purpose of providing clear views on the roles and functions of qualitative evidence in the process of guideline development.

## Methods

The systematic review was performed according to the PRISMA (Preferred Reporting Items for Systematic Reviews and Meta-Analysis) guidelines [14].

### Criteria for guideline selection

We included guidelines focused on improving healthcare that met the following criteria: 1) the guidelines were primarily published in Chinese or English from January 1, 2011 to February 25, 2020. In 2011, IOM (Institute of Medicine) claimed that for a CPG to be trustworthy it needs to “be developed via a transparent process by a group of multidisciplinary experts (including patient representatives), screened for minimal potential bias and conflicts of interest, and supported by a systematic review of the evidence” [15]. This, which is the first statement of criteria for clinical practice guidelines, plays an important role in guideline development, so we chose it as the start date for retrieval; 2) the guidelines met the above mentioned IOM criteria; 3) the guidelines mainly focused on clinical questions, such as diagnosis, treatment or care for certain diseases or patients symptoms, to provide suggestions for healthcare staff or community health services; 4) qualitative research or qualitative evidence was used in the process of guidelines development; 5) if the guidelines were updated, only the most recent version of the guidelines were included. The guidelines were excluded, if they had the following characteristics: 1) the same guidelines had been repeatedly published in multiple journals; 2) the full texts of guidelines were not available.

### Search strategy for guidelines

**Fig. 1:**
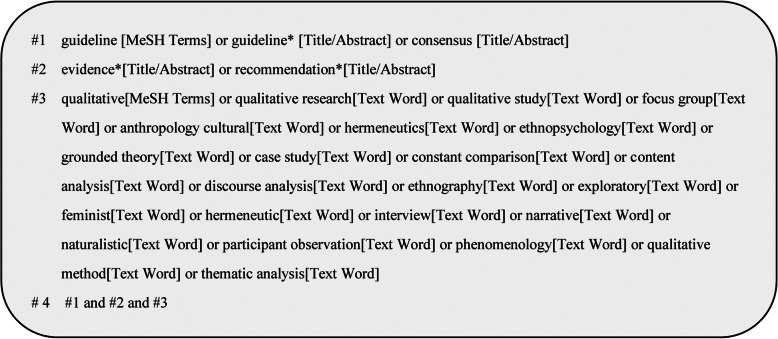
Search strategy on PubMed

Relevant representative guidelines repositories, such as WHO, NICE (the National Institute for Health and Care Excellence), SIGN (Scottish Intercollegiate Guidelines Network), NGC (National Guideline Clearinghouse), RNAO (Registered Nurses’ Association of Ontario), and other databases, including three English databases (PubMed, Embase, Web of Science), four Chinese databases (China National Knowledge Infrastructure, CNKI; Wanfang Data; Chinese BioMedical Literature Database, CBM; and VIP Database for Chinese Technical Periodicals, VIP), were systematically searched from January 1, 2011 to February 25, 2020. The search strategy used MeSH terms, Title/Abstract and text words. Taking PubMed as an example, the retrieval strategy is shown in Fig. 1.

### Guidelines selection and data extraction

Three (C.L.,Y.X.S and J.Z) authors experienced in literature retrieval independently selected eligible guidelines. Three reviewers (D.D.L.,Y.C and C.F) extracted significant information from the guidelines and completed data extraction forms by means of reading the text content of the guideline, references and the online relevant attachments. The detailed process of data extraction is presented in Additional file 1. The forms included: (1) the basic characteristics of included guidelines (such as title, publication/update date, and developer); (2) how qualitative research or evidence was used in the process of the guidelines development (were experts proficient in qualitative research invited to be involved in guideline development group, was qualitative research used to identify clinical questions, was qualitative evidence retrieved; was this used to support recommendations; and was this applied when considering facilitators and barriers to recommendations’ implementation); (3) details of the methodology for qualitative research or evidence used in the development process of guidelines (such as qualitative research quality assessment tool, the quality of the primary qualitative research study used to formulate recommendations and the grade of recommendations supported by qualitative evidence).

We hypothesized that the development of guidelines using qualitative research or evidence would be relevant to these items in the forms. The hypothesis was based on related methodological literature, COnsolidated criteria for REporting Qualitative research (COREQ) checklists [16] and discussion between all authors with methodologists in evidence-based guidelines development who were willing to engage in dialogue with us. Another researcher (Y.H.J) examined the data extraction forms to make sure no errors had occurred.

### Appraisal of included guidelines

Two researchers (Y.YW and D.H) independently evaluated the quality of the guidelines by using the Appraisal of Guidelines for Research and Evaluation (AGREE II) tool, which consists of 23 items under 6 domains involving scope and purpose, stakeholder involvement, rigor of development, clarity of presentation, applicability, and editorial independence [17]. Each item was rated from 1 to 7 points with 1 point for “strongly disagree” and 7 points for “strongly agree”. We summarized the domain scores individually and scaled the total of that domain, calculated by the following formula: (obtained score - minimal possible score)/(maximal possible score - minimal possible score) × 100% [17].

### Statistical analyses

Descriptive statistics were computed for the scores for each AGREE domain. Data for each AGREE II domain were provided as medians and interquartile ranges (IQRs). Intraclass correlation coefficients (ICCs) were calculated to evaluate the agreement between two reviewers for each domain [18, 19]. When the ICC value was less than 0.4, the consistency between raters was poor; if the ICC range was from 0.4 ~ 0.75, the consistency between raters was moderate; and a value of ICC over 0.75 the consistency was high [20]. The data were analyzed using SPSS version 17.0 (SPSS Inc. Chicago, IL, USA) and R version 3.3.2 (R Foundation for Statistical Computing, Vienna, Austria) for Windows.

## Results

### Guideline identification and selection

**Fig. 2:**
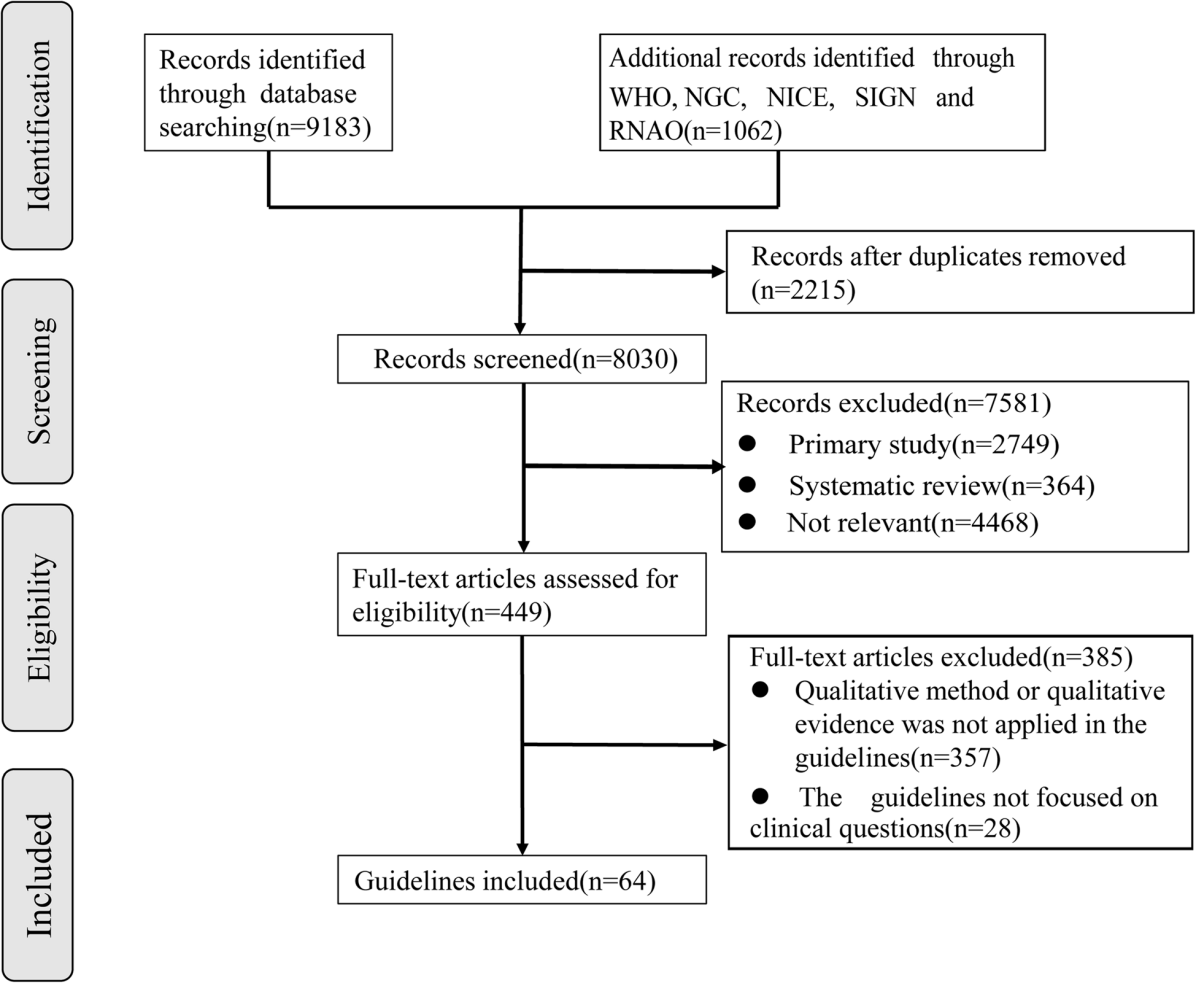
Flow diagram of guidelines identification and selection

The searches identified 10,245 discrete records, of which 449 were selected for a full-text review. Sixty-four guidelines were eventually included [21–84]. The flow diagram for the guidelines is shown in Fig. 2.

### Characteristics of included guidelines

As Table 1 shows, the sixty-four guidelines concentrated on different topics such as cancers, chronic pain and smoking, and were developed by NICE, SIGN, RNAO, WHO or other professional organizations. The majority of guideline developers used GRADE (the Grading of Recommendations Assessment, Development and Evaluation) criteria for grading of evidence and recommendations. When formulating recommendations, they considered the quality of evidence, the risk-benefit analysis of some interventions, supporting resources and stakeholders’ values and preferences. The number of recommendations ranged from 2 to 262. The largest number of recommendations supported only by qualitative evidence in each included guideline was 8 [68]. The largest number of recommendations supported by both qualitative and quantitative evidence in each included guideline was 23 [70]. The majority of recommendations were supported by qualitative evidence based on primary studies, a few on systematic reviews).

**Table 1:** The basic characteristics of guidelines included

No.	Title	Publication /updated (year)	Publishing organization	Grade of evidence and recommendation	Topic	Factors need to consider when formulating recommendations	Number of recommendations	The number of recommendations only supported by qualitative evidence	The number of recommendations supported by qualitative and quantitative evidence	The quantity and type of qualitative evidence	Update plan (period, organization, update criteria)	Number of references
1	Management of epithelial ovarian cancer [21]	2013/2018	SIGN	grade criteria developed by SIGN	epithelial ovarian cancer	the strength of the evidence;applicable; consistency of results	66	0	4	4, primary studies	3, GPAG, new evidence substantially changes a small number of recommendations, a specific issue (such as a new drug therapy or national issue), the nature of the update may not warrant assembling a multidisciplinary group	68
2	Diagnosis and management of epilepsy in adults [22]	2015/2018	SIGN	grade criteria developed by SIGN	epilepsy in adults	the strength of the evidence;applicable; consistency of results	224	0	3	3, primary studies	3, GPAG, new evidence substantially changes a small number of recommendations, a specific issue (such as a new drug therapy or national issue), the nature of the update may not warrant assembling a multidisciplinary group	104
3	Management of stable angina [23]	2018/−	SIGN	grade criteria developed by SIGN	stable angina	the quality (level) of the evidence;relevance to the NHS in Scotland; applicability of published evidence to the target population; consistency of the body of evidence, and the balance of benefits and harms of the options.	59	0	2	2, primary studies	3, GPAG, new evidence substantially changes a small number of recommendations, a specific issue (such as a new drug therapy or national issue), the nature of the update may not warrant assembling a multidisciplinary group	76
4	Cardiac rehabilitation [24]	2017/−	SIGN	grade criteria developed by SIGN	cardiac rehabilitation	the quality (level) of the evidence;relevance to the NHS in Scotland; applicability of published evidence to the target population; consistency of the body of evidence, and the balance of benefits and harms of the options.	36	0	5	5, primary studies systematic review	3, GPAG, new evidence substantially changes a small number of recommendations, a specific issue (such as a new drug therapy or national issue), the nature of the update may not warrant assembling a multidisciplinary group	49
5	Assessment, diagnosis and interventions for autism spectrum disorders [25]	2016/−	SIGN	grade criteria developed by SIGN	autism spectrum disorders	the quality (level) of the evidence;relevance to the NHS in Scotland; applicability of published evidence to the target population; consistency of the body of evidence, and the balance of benefits and harms of the options.	94	0	4	4, primary studies	3, GPAG, new evidence substantially changes a small number of recommendations, a specific issue (such as a new drug therapy or national issue), the nature of the update may not warrant assembling a multidisciplinary group	83
6	Management of chronic heart failure [26]	2016/−	SIGN	grade criteria developed by SIGN	chronic heart failure	the quality (level) of the evidence;relevance to the NHS in Scotland; applicability of published evidence to the target population; consistency of the body of evidence, and the balance of benefits and harms of the options.	80	0	3	3, primary studies	3, GPAG, new evidence substantially changes a small number of recommendations, a specific issue (such as a new drug therapy or national issue), the nature of the update may not warrant assembling a multidisciplinary group	82
7	Acute coronary syndrome [27]	2016/−	SIGN	grade criteria developed by SIGN	acute coronary syndrom	the quality (level) of the evidence;relevance to the NHS in Scotland; applicability of published evidence to the target population; consistency of the body of evidence, and the balance of benefits and harms of the options.	68	0	1	1, primary study	3, GPAG, new evidence substantially changes a small number of recommendations, a specific issue (such as a new drug therapy or national issue), the nature of the update may not warrant assembling a multidisciplinary group	66
8	British guideline on the management of asthma [28]	2016/−	SIGN	grade criteria developed by SIGN	asthma	the strength of the evidence, applicable, consistency of results	262	0	4	4, primary studies systematic review	3, GPAG, new evidence substantially changes a small number of recommendations, a specific issue (such as a new drug therapy or national issue), the nature of the update may not warrant assembling a multidisciplinary group	214
9	Glaucoma referral and safe discharge [29]	2015/−	SIGN	grade criteria developed by SIGN	glaucoma	the quality (level) of the evidence;relevance to the NHS in Scotland; applicability of published evidence to the target population; consistency of the body of evidence, and the balance of benefits and harms of the options.	60	0	3	3, primary studies systematic review	3, GPAG, new evidence substantially changes a small number of recommendations, a specific issue (such as a new drug therapy or national issue), the nature of the update may not warrant assembling a multidisciplinary group	45
10	Brain injury rehabilitation in adults [30]	2013/−	SIGN	grade criteria developed by SIGN	brain injury rehabilitation in adults	the strength of the evidence;applicable; consistency of results	54	0	1	1, primary study	3, GPAG, new evidence substantially changes a small number of recommendations, a specific issue (such as a new drug therapy or national issue), the nature of the update may not warrant assembling a multidisciplinary group	75
11	Management of hepatitis C [31]	2013/−	SIGN	grade criteria developed by SIGN	hepatitis C	the strength of the evidence;applicable; consistency of results	157	0	1	1, primary study	3, GPAG, new evidence substantially changes a small number of recommendations, a specific issue (such as a new drug therapy or national issue), the nature of the update may not warrant assembling a multidisciplinary group	64
12	Management of chronic pain [32]	2013/−	SIGN	grade criteria developed by SIGN	chronic pain	the strength of the evidence;applicable; consistency of results	64	0	1	1, systematic review	3, GPAG, new evidence substantially changes a small number of recommendations, a specific issue (such as a new drug therapy or national issue), the nature of the update may not warrant assembling a multidisciplinary group	71
13	Management of adult testicular germ cell tumours [33]	2011/−	SIGN	grade criteria developed by SIGN	adult testicular germ cell tumours	the strength of the evidence;applicable; consistency of results	97	0	2	2, primary studies	3, GPAG, new evidence substantially changes a small number of recommendations, a specific issue (such as a new drug therapy or national issue), the nature of the update may not warrant assembling a multidisciplinary group	70
14	Diagnosis and management of colorectal cancer [34]	2011/−	SIGN	grade criteria developed by SIGN	colorectal cancer	the strength of the evidence;applicable; consistency of results	114	0	1	1, primary study	3, GPAG, new evidence substantially changes a small number of recommendations, a specific issue (such as a new drug therapy or national issue), the nature of the update may not warrant assembling a multidisciplinary group	63
15	Implementing supervised injection services [35]	2018/−	RNAO	Adapted from SIGN and Pati D. A framework	injection services	benefits and harms,values and preferences,applicable, supporting resources	10	0	8	8, primary studies	5, IABPG, three months prior to the review milestone, new systematic reviews, randomized controlled trials, and other relevant literature in the field	108
16	Promoting and supporting the initiation, exclusivity, and continuation of breastfeeding for newborns, infants, and young children [36]	2018/−	RNAO	Adapted from SIGN and Pati D. A framework	breastfeeding for newborns, infants,and young children	benefits and harms,values and preferences,applicable,supporting resources	16	0	4	4, primary studies	5, IABPG, three months prior to the review milestone, new systematic reviews, randomized controlled trials, and other relevant literature in the field	204
17	Adult asthma care: promoting control of asthma second edition [37]	2004/2017	RNAO	Adapted from SIGN and Pati D. A framework	adult asthma care	benefits and harms,values and preferences,applicable, supporting resources	22	0	2	2, primary studies	5, IABPG, three months prior to the review milestone, new systematic reviews, randomized controlled trials, and other relevant literature in the field	128
18	Crisis intervention for adults using a trauma-informed Approach: initial four weeks of management third edition [38]	2002/2017	RNAO	Adapted from SIGN and Pati D. A framework	crisis intervention for adults	benefits and harms,values and preferences,applicable, supporting resources	13	0	2	2, primary studies	5, IABPG, three months prior to the review milestone, new systematic reviews, randomized controlled trials, and other relevant literature in the field	108
19	Delirium, dementia, and depression in older adults: assessment and care second edition [39]	2010/2016	RNAO	Adapted from SIGN and Pati D. A framework	delirium, dementia, and depression in older adults	benefits and harms,values and preferences,applicable, supporting resources	44	0	3	3, primary studies systematic review	5, IABPG, three months prior to the review milestone, new systematic reviews, randomized controlled trials, and other relevant literature in the field	164
20	Person- and family-centred care [40]	2015/−	RNAO	Adapted from SIGN and Pati D. A framework	person- and family-centred care	benefits and harms,values and preferences,applicable, supporting resources	15	0	6	6, primary studies systematic review	5, IABPG, three months prior to the review milestone, new systematic reviews, randomized controlled trials, and other relevant literature in the field	106
21	Care transitions [41]	2014/−	RNAO	Adapted from SIGN	care transitions	benefits and harms,values and preferences,applicable, supporting resources	22	0	4	4, primary studies	5, IABPG, three months prior to the review milestone, new systematic reviews, randomized controlled trials, and other relevant literature in the field	93
22	Preventing and addressing abuse and neglect of older adults: person-centred, collaborative, system-wide approaches [42]	2014/−	RNAO	Adapted from SIGN and Pati D. A framework	abuse and neglect of older adults	benefits and harms,values and preferences,applicable, supporting resources	22	0	5	6, primary studies	5, IABPG, three months prior to the review milestone, new systematic reviews, randomized controlled trials, and other relevant literature in the field	130
23	Primary prevention of childhood obesity second edition [43]	2005/2014	RNAO	Adapted from SIGN	childhood obesity	benefits and harms,values and preferences,applicable, supporting resources	21	0	2	2, primary studies	5, IABPG, three months prior to the review milestone, new systematic reviews, randomized controlled trials, and other relevant literature in the field	144
24	Assessment and management of foot ulcers for people with diabetes second edition [44]	2005/2013	RNAO	Adapted from SIGN	foot ulcers for people with diabetes	benefits and harms,values and preferences,applicable, supporting resources	27	**2**	**1**	4, primary study	5, IABPG, three months prior to the review milestone, new systematic reviews, randomized controlled trials, and other relevant literature in the field	160
25	Promoting safety: alternative approaches to the use of restraints [45]	2012/−	RNAO	Adapted from SIGN	promoting safety	benefits and harms,values and preferences,applicable, supporting resources	12	0	7	7, primary studies systematic review	5, IABPG, three months prior to the review milestone, new systematic reviews, randomized controlled trials, and other relevant literature in the field	152
26	Depression in children and young people: identification and management [46]	2019	NICE	GRADE	depression in children and young people	the evidence available,the individual needs,preferences and values of patients	121	0	1	1, primary studies	3, NICE’ s Guidance Executive, references to other NICE guidance or hyperlinks to other NICE endorsed tools or resources, the latest government policy or guidelines, reflect the current practice context	44
27	Pancreatic cancer in adults: diagnosis and management [47]	2018/−	NICE	GRADE	pancreatic cancer in adults	the evidence available,the individual needs,preferences and values of patients	57	0	1	1, primary study	3, NICE’ s Guidance Executive, references to other NICE guidance or hyperlinks to other NICE endorsed tools or resources, the latest government policy or guidelines, reflect the current practice context	21
28	Antimicrobial stewardship: changing risk related behaviours in the general population [48]	2017/−	NICE	GRADE	antimicrobial stewardship	the evidence available,the individual needs,preferences and values of patients	35	0	1	2, primary studies	3, NICE’ s Guidance Executive, references to other NICE guidance or hyperlinks to other NICE endorsed tools or resources, the latest government policy or guidelines, reflect the current practice context	44
29	Eating disorders: recognition and treatment [49]	2017/−	NICE	GRADE	eating disorders	the evidence available,the individual needs,preferences and values of patients	139	0	1	1, primary study	3, NICE’ s Guidance Executive, references to other NICE guidance or hyperlinks to other NICE endorsed tools or resources, the latest government policy or guidelines, reflect the current practice context	41
30	Healthcare-associated infections: prevention and control in primary and community care [50]	2012/2017	NICE	GRADE	healthcare-associated infections	the evidence available,the individual needs,preferences and values of patients	102	1	0	1, primary study	3, NICE’ s Guidance Executive, references to other NICE guidance or hyperlinks to other NICE endorsed tools or resources, the latest government policy or guidelines, reflect the current practice context	33
31	Hip fracture: management [51]	2011/2017	NICE	GRADE	hip fracture	the evidence available,the individual needs,preferences and values of patients	33	0	2	2, primary studies	3, NICE’ s Guidance Executive, references to other NICE guidance or hyperlinks to other NICE endorsed tools or resources, the latest government policy or guidelines, reflect the current practice context	19
32	Immunisations: reducing differences in uptake in under 19 s [52]	2009/2017	NICE	GRADE	immunisations	the evidence available,the individual needs,preferences and values of patients	6	0	2	60, primary studies systematic review	3, NICE’ s Guidance Executive, references to other NICE guidance or hyperlinks to other NICE endorsed tools or resources, the latest government policy or guidelines, reflect the current practice context	60
33	Intermediate care including reablement [53]	2017/−	NICE	GRADE	intermediate care	the evidence available,the individual needs,preferences and values of patients	52	0	5	5, primary studies	3, NICE’ s Guidance Executive, references to other NICE guidance or hyperlinks to other NICE endorsed tools or resources, the latest government policy or guidelines, reflect the current practice context	28
34	Suspected cancer: recognition and referral [54]	2015/2017	NICE	GRADE	suspected cancer	the evidence available,the individual needs,preferences and values of patients	110	0	1	1, primary study	3, NICE’ s Guidance Executive, references to other NICE guidance or hyperlinks to other NICE endorsed tools or resources, the latest government policy or guidelines, reflect the current practice context	79
35	Coexisting severe mental illness and substance misuse: community health and social care services [55]	2016/−	NICE	GRADE	coexisting severe mental illness and substance misuse	the evidence available,the individual needs,preferences and values of patients	50	6	4	153, primary studies systematic review	3, NICE’ s Guidance Executive, references to other NICE guidance or hyperlinks to other NICE endorsed tools or resources, the latest government policy or guidelines, reflect the current practice context	60
36	Oral health for adults in care homes [56]	2016/−	NICE	GRADE	adults in care homes	the evidence available,the individual needs,preferences and values of patients	22	0	11	93, primary studies	3, NICE’ s Guidance Executive, references to other NICE guidance or hyperlinks to other NICE endorsed tools or resources, the latest government policy or guidelines, reflect the current practice context	37
37	Skin cancer prevention [57]	2011/2016	NICE	GRADE	skin cancer	the evidence available,the individual needs,preferences and values of patients	6	1	4	54, primary studies	3, NICE’ s Guidance Executive, references to other NICE guidance or hyperlinks to other NICE endorsed tools or resources, the latest government policy or guidelines, reflect the current practice context	64
38	Maternal and child nutrition [58]	2008/2014	NICE	GRADE	maternal and child nutrition	the evidence available,the individual needs,preferences and values of patients	15	0	4	4, primary studies	3, NICE’ s Guidance Executive, references to other NICE guidance or hyperlinks to other NICE endorsed tools or resources, the latest government policy or guidelines, reflect the current practice context	98
39	Needle and syringe programmes [59]	2014/−	NICE	GRADE	needle and syringe programmes	the evidence available,the individual needs,preferences and values of patients	10	1	3	7, primary studies	3, NICE’ s Guidance Executive, references to other NICE guidance or hyperlinks to other NICE endorsed tools or resources, the latest government policy or guidelines, reflect the current practice context	58
40	Physical activity: brief advice for adults in primary care [60]	2013/−	NICE	GRADE	adults in primary care	the evidence available,the individual needs,preferences and values of patients	5	3	2	68, primary studies	3, NICE’ s Guidance Executive, references to other NICE guidance or hyperlinks to other NICE endorsed tools or resources, the latest government policy or guidelines, reflect the current practice context	60
41	Service user experience in adult mental health: improving the experience of care for people using adult NHS mental health services [61]	2011/−	NICE	GRADE	adult mental health	the evidence available,the individual needs,preferences and values of patients	44	0	5	7, primary studies	3, NICE’ s Guidance Executive, references to other NICE guidance or hyperlinks to other NICE endorsed tools or resources, the latest government policy or guidelines, reflect the current practice context	42
42	Type 2 diabetes prevention: population and community-level interventions [62]	2011/−	NICE	GRADE	type 2 diabetes prevention	the evidence available,the individual needs,preferences and values of patients	11	3	4	67, primary studies	3, NICE’ s Guidance Executive, references to other NICE guidance or hyperlinks to other NICE endorsed tools or resources, the latest government policy or guidelines, reflect the current practice context	84
43	WHO recommendations Intrapartum care for a positive childbirth experience [63]	2018	WHO	GRADE	Intrapartum care	the evidence domains on values, Equity, acceptability and feasibility	56	0	12	15, primary studies systematic review	updated after five years as more evidence becomes available	210
44	Guidelines for managing advanced HIV disease and rapid initiation of antiretroviral therapy [64]	2017	WHO	GRADE	HIV disease	the evidence domains on values, Equity, acceptability and feasibility	2	0	1	5, primary studies	updated after five years as more evidence becomes available	56
45	Protecting, promoting and supporting breastfeeding in facilities providing maternity and newborn services [65]	2017	WHO	GRADE	breastfeeding	the evidence domains on values, Equity, acceptability and feasibility	15	6	6	42, primary studies	updated after five years as more evidence becomes available	136
46	Guidelines on HIV self-testing and partner notification: supplement to consolidated guidelines on HIV testing services [66]	2016	WHO	GRADE	HIV self-testing and partner notification	the evidence domains on values, Equity, acceptability and feasibility	12	0	4	10, primary studies systematic review	updated after five years as more evidence becomes available	104
47	WHO recommendations on antenatal care for a positive pregnancy experience [67]	2016	WHO	GRADE	antenatal care	the evidence domains on values, Equity, acceptability and feasibility	49	6	6	10, primary studies systematic review	updated after five years as more evidence becomes available	172
48	Health worker roles in providing safe abortion care and post-abortion contraception [68]	2015	WHO	GRADE	safe abortion care and post-abortion contraception	the evidence domains on values, Equity, acceptability and feasibility	116	8	8	204, primary studies systematic review	updated after five years as more evidence becomes available	92
49	WHO recommendations on health promotion interventions for maternal and newborn health [69]	2015	WHO	GRADE	maternal and newborn health	the evidence domains on values, Equity, acceptability and feasibility	12	0	6	6, primary studies systematic review	updated after five years as more evidence becomes available	94
50	WHO recommendations optimizing health worker roles to improve access to key maternal and newborn health interventions through task shifting [70]	2012	WHO	GRADE	maternal and newborn health interventions	the evidence domains on values, Equity, acceptability and feasibility	38	0	23	17, primary studies systematic review	updated after five years as more evidence becomes available	98
51	Nursing practice guideline for emergency percutaneous coronary intervention [71]	2019	NCCD, CCBNA, RC-NTPC-AMSPUMC, EBMCLU	GRADE	emergency percutaneous coronary intervention	–	20	–	–	–	–	6
52	Expert consensus on breast tumor plastic surgery and breast reconstruction (2018 edition) [72]	2018/−	CBCS, CSBS	GRADE	breast cancer	–	46	0	3	3, primary studies	–	42
53	Gestational diabetes mellitus clinical nursing practice guideline [73]	2018	MHFU, SNFU, SEBNC	GRADE	Gestational diabetes mellitus	–	69	0	1	2, primary studies systematic review	updated every three to five years	116
54	Evidence-based guidelines for breastfeeding of hospitalized newborns [74]	2017	PHFU, SNFU, JBI-EBNCCFU, SEBNC	GRADE	breastfeeding of hospitalized newborns	–	83	–	–	–	updated every three to five years	97
55	Clinical nursing guideline on cancer related fatigue in adults [75]	2017	JBI-EBNCCFU	GRADE	cancer related fatigue in adults	–	33	–	–	–	–	5
56	Clinical application of anaesthesia in accelerated rehabilitation surgery of colorectal surgery in lingnan expert consensus on operation specification (2016 edition) [76]	2016/−	GD-MAARSB	GRADE	enterosurgery	–	35	0	2	2, systematic review	–	11
57	HIV/AIDS nursing clinical practice guidelines [77]	2016	SPHCC, JBI-EBNCCFU	GRADE	HIV/AIDS nursing	–	139	0	2	2, systematic review	updated every three to five years	216
58	Nursing practice guideline of acute heart failure [78]	2016	NCCD, HFCCMA, BNS	GRADE	acute heart failure	–	25	–	–	–	–	10
59	Clinical nursing practice guideline for enteral nutrition for infants with congenital heart disease [79]	2016	JBI-EBNCCFU, SEBNC, PHFU	GRADE	congenital heart disease	–	38	–	–	–	updated every five years	134
60	Clinical practice guideline for nasogastric tube feeding among adult patients [80]	2015	ECHFU, SNFU	GRADE	nasogastric tube feeding among adult patients	–	99	–	–	–	–	142
61	Clinical practice guidelines of peripherally inserted central catheter (PICC) catheterization [81]	2014	FUSCC, SNFU, JBI-EBNCCFU	GRADE	peripherally inserted central catheter (PICC) catheterization	–	56	0	1	1, primary studies	updated every three to five years	66
62	Evidence-based clinical practice guideline on prevention and management of medication errors in hospitalized adult patients [82]	2014	SNFU	GRADE	prevention and management of medication errors	–	28	–	–	–	–	4
63	Clinical practice guideline for oral care on critically ill patients with endotracheal intubation [83]	2013	JBI-EBNCCFU, SNFU	GRADE	oral care on critically ill patients	–	17	–	–	–	–	110
64	Clinical practice guideline on inpatient fall prevention [84]	2011	JBI-EBNCCFU	GRADE	inpatient fall prevention	–	31	–	–	–	updated every three to five years	57

*SIGN* Scottish Intercollegiate Guidelines Network, *RNAO* Registered Nurses’ Association of Ontario, *NICE* the National Institute for Health and Care Excellence, *GD-MAARSB* Guangdong Provincial Medical Association Accelerated Rehabilitation Surgeons Branch, *CBCS* Committee of Breast Cancer Society, *CSBS* Committee Specialist of Breast Surgeons, *GRADE* Grading of Recommendations Assessment, Development, and Evaluation, *GPAG* the Guideline Programme Advisory Group, *IABPG* International Affairs and Best Practice Guideline, *NCCD* National Center for Cardiovascular Diseases, *CCBNA* Cardialvascular Committee of Beijing Nursing Assossiation, *RC-NTPC-AMSPUMC* Research Center of Nursing Theory and Practice Chinese Academy of Medical Sciences &Peking Union Medical College, *EBMCLU* Evidence-based Medical Center of Lanzhou University, *MHFU* Maternity Hospital of Fudan University, *SNFU* School of Nursing, Fudan University, *SEBNC* Shanghai Evidence-based Nursing Center, *PHFU* Pediatric Hospital of Fudan University, *JBI-EBNCCFU* JBI Evidence-based Nursing Cooperation Center of Fudan University, *SPHCC* Shanghai Public Health Clinical Center, *HFCCMA* Heart Failure Committee of Chinese Medical Association, *BNS* Beijing Nursing Society, *ECHFU* East China Hospital of FudanUniversity, *FUSCC* Fudan University Shanghai Cancer Center

### Quality appraisal of the guidelines

The ICC values for all six domains were over 0.75, which indicated high consistency in the assessment results between the two raters.

**Fig. 3:**
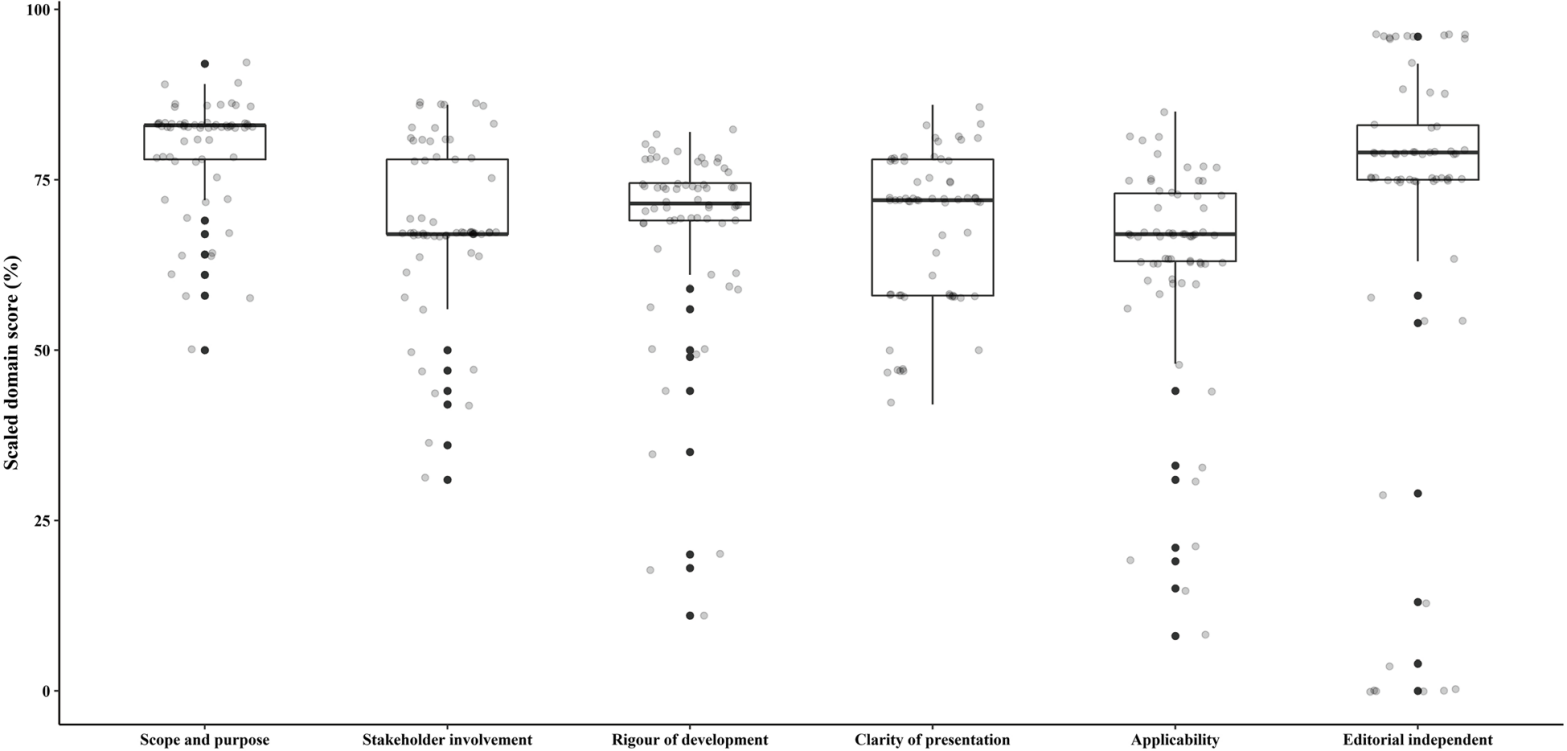
The summary of scaled domain score over all included guidelines

As Table 2 and Fig. 3 show. The final domain scores ranged between 0% (domain 6 of 6 guidelines) [75, 77, 78, 81, 82, 84] and 96% (domain 6 of 11 guidelines) [21, 22, 25–27, 29–34]. When comparing the total domain scores, Domain 1 (Scope and Purpose) was ranked the highest with a median score of 83% (IQ 78–83). Domain 2 (Stakeholder involvement) and Domain 5 (Applicability) were ranked the lowest with median scores of 67% (IQ 67–78) and 67% (IQ 63–73) respectively. The median scores of Domains 3, 4, 6 (Rigour of development, Clarity of presentation, Editorial independence) were 71% (IQ 69–74), 72% (IQ 58–78) and 79% (IQ 75–83) respectively.

**Table 2:** Analysis of the included N-CPGs according to AGREE II (%)

Guidelines	Scope and purpose	Stakeholder involvement	Rigour of development	Clarity of presentation	Applicability	Editorial independence
1	83	64	74	72	63	96
2	83	67	74	72	63	96
3	83	67	74	72	63	79
4	83	67	74	72	63	79
5	78	67	74	72	63	96
6	83	67	74	72	63	96
7	83	67	74	72	63	96
8	83	67	74	72	63	79
9	83	67	74	72	63	96
10	78	67	74	72	63	96
11	78	67	74	72	63	96
12	78	69	74	72	63	96
13	83	64	74	72	60	96
14	83	69	74	72	60	96
15	89	86	78	78	77	79
16	83	83	78	78	77	79
17	86	75	80	78	73	79
18	83	83	78	75	81	79
19	83	81	79	78	73	79
20	86	78	79	75	77	79
21	89	86	78	728	73	79
22	83	81	78	78	79	79
23	86	81	77	72	75	75
24	86	86	78	72	73	79
25	86	83.	76	78	73	79
26	72	78	65	72	60	88
27	78	67	69	47	67	75
28	83	67	69	58	67	75
29	83	67	69	58	67	75
30	83	67	69	58	67	75
31	83	67	71	58	67	75
32	83	67	69	42	67	75
33	83	67	71	58	67	75
34	83	67	71	58	67	75
35	83	67	69	58	67	75
36	83	67	69	58	67	75
37	83	67	69	47	67	75
38	83	67	71	47	67	75
39	83	67	71	58	67	75
40	83	67	69	58	67	75
41	83	67	71	58	67	75
42	83	67	69	47	67	75
43	78	78	82	83	81	75
44	83	78	61	86	75	54
45	92	86	82	78	81	88
46	86	78	50	81	75	63
47	86	86	78	83	85	92
48	83	64	78	81	71	88
49	81	81	70	81	75	58
50	81	86	78	78	71	54
51	64	47	59	75	19	79
52	78	56	18	47	21	4
53	64	67	72	67	75	79
54	58	69	50	78	33	83
55	61	58	35	58	15	0
56	67	50	20	50	67	13
57	72	42	44	81	48	0
58	72	36	61	81	60	0
59	75	81	72	72	60	83
60	81	31	71	75	58	29
61	64	81	56	64	56	0
62	50	61	11	50	8	0
63	69	47	49	67	44	83
64	58	44	59	61	31	0
Median, interquartile range (25, 75%)	83 (78, 83)	67 (67, 78)	71(69, 74)	72 (58, 78)	67(63, 73)	79(75, 83)

### The process of the guidelines development using qualitative research or evidence

**Fig. 4:**
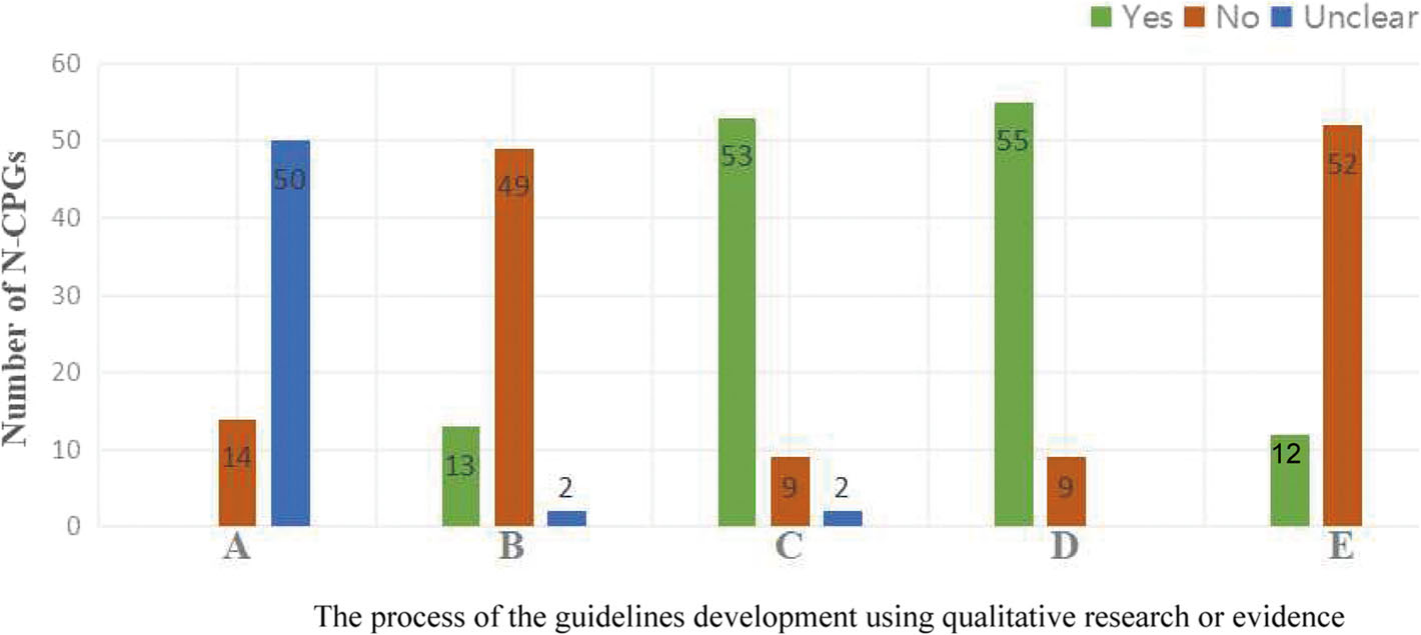
The process of the guidelines development using qualitative research or evidence. **a** Experts proficient in qualitative research to involve in guideline development group. **b** Using qualitative research to identify clinical questions. **c** Retrieving qualitative evidence. **d** Using qualitative evidence to support recommendations. **e** Applying qualitative evidence when considering facilitators and barriers of recommendations' implementation

As Fig. 4 shows, no guideline developers invited experts proficient in qualitative research to be involved in guideline development groups. 20% guidelines (13/64) used qualitative research to identify clinical questions [68, 71, 73–75, 77–84]. 83% (53/64) guidelines retrieved qualitative evidence [21–70, 75, 77, 81]. 86% (55/64) guidelines used qualitative evidence to support recommendations [21–70, 72, 75–77, 81]. And 19% (12/64) guidelines applied qualitative evidence when considering facilitators and barriers to recommendations’ implementation [55, 56, 60, 62–70].

### The methodology for evidence used in the guidelines development

As Table 3 shows, one guideline used qualitative research based on grounded theory, phenomenology [55]. 52% (27/52) guideline developers evaluated the quality of the primary qualitative research study using the CASP (the Critical Appraisal Skills Programme) tool or NICE checklist for qualitative studies [35, 38, 46–70]. No guidelines evaluated (0/18) the quality of qualitative evidence synthesis used to formulate recommendations. 17% (11/64) guidelines presented the level of qualitative research using the grade criteria of evidence and recommendation in different forms such as I, III, IV, very low [35–40, 42, 44, 73, 77, 81]. They were based on JBI, GRADE or adapted from SIGN or Pati D. A framework [35–45, 85–87] respectively. 28% guidelines (15/54) described the grades of the recommendations supported by qualitative and quantitative evidence in different ways such as “strong”, “good”, “B”, “C” or “D” and “weak” [21, 22, 24, 25, 27, 28, 30–34, 73, 76, 77, 81], which also complied with JBI, GRADE or adapted from SIGN and (or) Pati D. A framework respectively. But no guidelines (0/10) described the grade of recommendations supported only by qualitative evidence.

**Table 3:** The methodology for qualitative research or evidence in the process of included guidelines development

No.	The theory basis of qualitative research	The quality assessment tool for qualitative research	The quality level of primary study of qualitative research to formulate recommendations	The quality level of qualitative evidence synthesis to formulate recommendations	The level of qualitative research in the grade criteria of evidence and recommendation	The grade of recommendations only supported by qualitative evidence	The grade of recommendations supported by qualitative and quantitative evidence
1	–	–	–	–	–	–	Good
2	–	–	–	–	–	–	B
3	–	–	–	–	–	–	–
4	–	–	–	–	–	–	Good
5	–	–	–	–	–	–	Strong
6	–	–	–	–	–	–	–
7	–	–	–	–	–	–	Strong
8	–	–	–	–	–	–	D
9	–	–	–	–	–	–	–
10	–	–	–	–	–	–	B
11	–	–	–	–	–	–	Good
12	–	–	–	–	–	–	Good
13	–	–	–	–	–	–	D
14	–	–	–	–	–	–	D
15	–	CASP	High: greater than, or equal to, a converted score of 82.4% Moderate: a converted score of 62.5–82.3%	–	III, IV^1)^	–	–
16	–	–	–	–	III, IV^1)^	–	–
17	–	–	–	–	III, IV^1)^	–	–
18	–	CASP	High: greater than, or equal to, a converted score of 82.4% Moderate: a converted score of 62.5–82.3%	–	III, IV^1)^	–	–
19	–	–	–	–	III, IV^1)^	–	–
20	–	–	–	–	III, IV^1)^	–	–
21	–	–	–	–	–	–	–
22	–	–	–	–	III, IV^1)^	–	–
23	–	–	–	–	–	–	–
24	–	–	–	–	III, IV^1)^	–	–
25	–	–	–	–	–	–	–
26	–	NICE checklist	–	–	–	–	–
27	–	NICE checklist	–	–	–	–	–
28	–	NICE checklist	–	–	–	–	–
29	–	NICE checklist	–	–	–	–	–
30	–	NICE checklist	–	–	–	–	–
31	–	NICE checklist	–	–	–	–	–
32	–	NICE checklist	–	–	–	–	–
33	–	NICE checklist	+: indicates that some of the checklist criteria have been fulfilled -: indicates that few or no checklist criteria have been fulfilled	–	–	–	–
34	–	NICE checklist	–	–	–	–	–
35	Grounded theory, phenomenology	NICE checklist	++: indicates that all or most of the checklist criteria have been fulfilled +: indicates that some of the checklist criteria have been fulfilled –: indicates that few or no checklist criteria have been fulfilled	–	–	–	–
36	–	NICE checklist	++: indicates that all or most of the checklist criteria have been fulfilled +: indicates that some of the checklist criteria have been fulfilled -: indicates that few or no checklist criteria have been fulfilled	–	–	–	–
37	–	NICE checklist	++: indicates that all or most of the checklist criteria have been fulfilled +: indicates that some of the checklist criteria have been fulfilled -: indicates that few or no checklist criteria have been fulfilled	–	–	–	–
38	–	NICE checklist	–	–	–	–	–
39	–	NICE checklist	+: indicates that some of the checklist criteria have been fulfilled −: indicates that few or no checklist criteria have been fulfilled	–	–	–	–
40	–	NICE checklist	++: indicates that all or most of the checklist criteria have been fulfilled +: indicates that some of the checklist criteria have been fulfilled -: indicates that few or no checklist criteria have been fulfilled	–	–	–	–
41	–	NICE checklist	++: indicates that all or most of the checklist criteria have been fulfilled +: indicates that some of the checklist criteria have been fulfilled	–	–	–	–
42	–	NICE checklist	++: indicates that all or most of the checklist criteria have been fulfilled +: indicates that some of the checklist criteria have been fulfilled -: indicates that few or no checklist criteria have been fulfilled	–	–	–	–
43	–	CASP	High: greater than, or equal to, a converted score of 82.4% Moderate: a converted score of 62.5–82.3%	–	–	–	–
44	–	CASP	High: greater than, or equal to, a converted score of 82.4% Moderate: a converted score of 62.5–82.3%	–	–	–	–
45	–	CASP	High: greater than, or equal to, a converted score of 82.4% Moderate: a converted score of 62.5–82.3%	–	–	–	–
46	–	CASP	High: greater than, or equal to, a converted score of 82.4% Moderate: a converted score of 62.5–82.3%	–	–	–	–
47	–	CASP	High: greater than, or equal to, a converted score of 82.4% Moderate: a converted score of 62.5–82.3%	–	–	–	–
48	–	CASP	High: greater than, or equal to, a converted score of 82.4% Moderate: a converted score of 62.5–82.3%	–	–	–	–
49	–	CASP	High: greater than, or equal to, a converted score of 82.4% Moderate: a converted score of 62.5–82.3%	–	–	–	–
50	–	CASP	High: greater than, or equal to, a converted score of 82.4% Moderate: a converted score of 62.5–82.3%	–	–	–	–
51	–	–	–	–	–	–	–
52	–	–	–	–	–	–	–
53	–	–	–	–	Very low	–	weak
54	–	–	–	–	–	–	–
55	–	–	–	–	–	–	–
56	–	–	–	–	–	–	Strong
57	–	–	–	–	I, IV^2)^	–	B
58	–	–	–	–	–	–	–
59	–	–	–	–	–	–	–
60	–	–	–	–	–	–	–
61	–	–	–	–	IV^2)^	–	B
62	–	–	–	–	–	–	–
63	–	–	–	–	–	–	–
64	–	–	–	–	–	–	–

CASP: the Critical Appraisals Skills Programme; III: Synthesis of multiple studies primarily of qualitative research; IV^1)^: Evidence obtained from well-designed non-experimental observational studies, such as analytical studies or descriptive studies, and/or qualitative studies; I: Evidence obtained from meta-analysis or systematic reviews of randomized controlled trials, and/or synthesis of multiple studies primarily of quantitative research; Evidence obtained from at least one randomized controlled trial; IV^2)^: Evidence obtained from well-designed non-experimental observational studies, such as analytical studies or descriptive studies, and/or qualitative studies. Very low: the guideline development group have very little confidence in the effect estimate, the true effect is likely to be substantially different from the estimate of effect; Good: Recommended best practice based on the clinical experience of the guideline development group; B: a body of evidence including studies rated as 2++, directly applicable to the target population, and demonstrating overall consistency of results; or extrapolated evidence from studies rated as 1++ or 1+; D: evidence level 3 or 4, or extrapolated evidence from studies rated as 2+; Strong: the guideline development group is confident that for the vast majority people, the intervention (or the interventions) will do more good than harm or do more harm than good; Weak: the guideline development group is uncertain about the advantages and disadvantages or high or low quality evidence shows that the advantages and disadvantages are equivalent

## Discussion

Our review shows that the majority of the included guidelines were high-quality. Qualitative evidence was mainly used to identify clinical questions, support recommendations, and consider facilitators and barriers to recommendations’ implementation. However, the methodology still needs more attention, as there were, no experts proficient in qualitative research involved in guideline development group, no assessment of the quality of qualitative evidence synthesis and a lack of detailed reporting the level of qualitative evidence and its grade of recommendations’.

### The summary findings of this review

The majority of the included guidelines introduced the overall aim of the guideline, the specific health questions, and the target population in tabulated form, bold, or using separate paragraphs. They described the gathering and synthesis of the evidence, gave details of updating and dealt with the language, structure, and format of the guideline recommendations.. However, the guidelines still had some noticeable shortcomings. For instance, a few guidelines did not describe the methods of formulating recommendations [74, 76, 82]; a few did not clearly introduce the different options for management of the conditions or health issues [76, 82]; a minority of guidelines did not give details of conflict of interest statements [75, 77, 78, 81, 82, 84]. In addition, although the majority of the guidelines stated that the guideline development group consisted of all relevant professional experts, and clearly defined the guidelines’ target users, a number of developers did not consider values and preferences of the target population [71, 78, 83, 84] or lacked adequate information on how they gained patients, doctors or other stakeholders’ views. And also the majority of the guidelines did not describe facilitators and barriers to their application in detail.

The methodological quality of qualitative evidence affects interpretation of its results. Unfortunately, while the majority of guidelines developers used qualitative evidence synthesis to formulate recommendations, they did not appraise confidence in each individual review, which resulted in some difficulties in explaining relevant themes or theories formulated in different articles. In addition, only three of the grade systems used, referred to single qualitative studies or synthesis of qualitative research as a level of the grade criteria of evidence and recommendation [35–45, 85–87]. The majority of guideline developers did not concentrate on the important influence of qualitative evidence on the grade criteria of evidence and recommendation.

### Comparison of findings with prior research

When comparing our findings with similar relevant articles, lack of statements about conflict of interest, details on how to gain patients, doctors or other stakeholders’ views, consideration of facilitators and barriers to guidelines’ implementation are also common issues e.g. oncology CPGs [88], inflammatory bowel disease guidelines [89], nursing CPGs [90], guidelines for management of cholangiocarcinoma [91]. Our review firstly identified whether qualitative research or evidence had been used to obtain stakeholders’ values and preferences, and in identifying facilitators and barriers to guidelines’ implementation in the process of guidelines development. Other researchers also used qualitative research to explore practice gaps based on existing guidelines: Feyissa et al. used a semi-structured interview to assess contextual barriers and facilitators to the implementation of a guideline developed to reduce HIV-related stigma and discrimination (SAD) in the Ethiopian healthcare setting [92]; Lind et al. interviewed local politicians, chief medical officers and health professionals at acute care hospitals to investigate perceptions regarding guidelines for palliative care and identify obstacles and opportunities for their implementation in acute care hospitals [93].

In Addition, qualitative research is increasingly being recognised as having an important role to play in addressing questions relating to interventions or system complexity, and guideline development processes. As with our topic, other researchers have also focused on the methodology of involving qualitative research in the development process of guidelines. Flemming et al. provided guidance for the choice of qualitative evidence synthesis methods in the context of guideline development for complex interventions by using a best fit framework synthesis to address interactions between components of complex interventions; interactions of interventions with context and multiple (health and non-health) outcomes; using meta-ethnography to deal with sociocultural acceptability of an intervention [94]. In addition, Moore et al. also put forward designs and methods for the applicability of quantitative and qualitative evidence in guidelines including complexity-related questions of interest in the guideline, types of synthesis used in the guideline, mixed-method review design and integration mechanisms, observations, concerns and considerations [95].

### Implications for guideline developers

The development of guidelines is a complex undertaking which needs a significant focus on its methodology. Based on our findings, we put forward some proposals for guideline developers, which may be helpful to improve their guideline’s quality. Firstly, guidelines developers can record and report details about how they reach agreement on recommendations and how they deal with possible disagreement when formulating recommendations and present different options for the same CQs with information on population characteristics or clinical situations for each option. Secondly, they can also develop a series of methods to avoid potential COI before the initiation of the guideline development project. Guideline developers may also obtain the target population’ views by interviewing stakeholders or extracting some relevant themes from existing qualitative data on the topic of interest. Finally, guideline developers should formally consider how to evaluate and grade single qualitative studies or synthesis of qualitative research into the grade system for guideline development prior to start-up of the guideline development project, and identify which factors influence the grade classification with the help of experts proficient in qualitative research. They should also select appropriate tools to appraise the quality of qualitative evidence such as CASP tool, NICE checklist for primary studies, GRADE-CERQual (Grading of Recommendations Assessment, Development and Evaluation-Confidence in the Evidence from Reviews of Qualitative research) for qualitative evidence synthesis, which is an approach for assessing how much confidence to place in findings from qualitative evidence syntheses in terms of four components (methodological limitations, coherence, adequacy of data, relevance) [13, 96].

### Limitations and strengths

Our study has some potential limitations. Firstly, although we selected eligible guidelines by means of reading their text content, references and the online relevant attachments, we used a quick search strategy on the guideline development. We also used the filter capability when using Endnote to manage literature from databases. But because of the size of the task there may be selection bias because of unavailable guidelines published in government documents, books or other guideline publication platforms. Additionally, we did not specify how many guidelines were recommended, recommended with modifications, and not recommended, because AGREE II protocol states that no overall score is calculated to determine if a CPG is recommended or not recommended and the main focus of this article was the methodology for qualitative research or qualitative evidence used in guidelines development [17]. Nonetheless, there may be several advantages. Firstly, a systematic literature search was performed for screening eligible guidelines. Secondly, we discussed the potential effect of qualitative research or evidence on the AGREE II appraisal, and then put forward some suggestions on how to use qualitative research or evidence to improve the quality of future guidelines. Thirdly, this is the first attempt to systematically analyze the role of qualitative research or evidence in guidelines development based on published guidelines.

### Suggestions for ongoing research

Qualitative research or qualitative evidence will be extensively used in the guideline development process in the future. There are three interesting topics needing further research. Firstly, when available data exists, this can be explored to provide more reliable conclusions related to the potential association between AGREE appraisal and the identification, incorporation and reporting of qualitative research by means of statistical methods such as non-parametric tests. Secondly, it will be interesting to compare the use of qualitative and quantitative data when formulating recommendations in guidelines, perhaps by matching guidelines on similar topics or key questions, and comparing those which did and didn’t use use qualitative evidence. Thirdly, exploring how qualitative research may be used to obtain the information related to conflict of interest will also be useful to inform guideline transparency. These topics are worthy of future exploration.

## Conclusion

The majority of the included guidelines were high-quality. Qualitative evidence was mainly used to identify clinical questions, support recommendations, and consider facilitators and barriers to recommendations’ implementation. However, more attention needs to be given to the methodology, for instance, no experts proficient in qualitative research have been involved in guideline development group, there has been no assessment of the quality of qualitative evidence synthesis, and there is a lack of detail when reporting on the level of qualitative evidence and its grade recommendations’.

## Supplementary information

**Additional file 1.** The process of data extraction.

## Data Availability

All data generated or analyzed are included in this published article.

## Abbreviations

WHO: World Health Organization
GRC: Guidelines Review Committee
CQ: Clinical Questions
SIGN: Scottish Intercollegiate Guidelines Network
NGC: National Guideline Clearinghouse
NICE: The National Institute for Health and Care Excellence
RNAO: Registered Nurses’ Association of Ontario
CNKI: China National Knowledge Infrastructure
CBM: Chinese BioMedical Literature Database
VIP: VIP Database for Chinese Technical Periodicals
AGREE II: The Appraisal of Guidelines for Research and Evaluation
IQR: Interquartile Range
ICC: Intraclass Correlation Coefficient
GRADE: The Grading of Recommendations Assessment, Development and Evaluation
ICU: Intensive Care Unit
CASP: The Critical Appraisal Skills Programme
FCC: Family-Centered Care
JBI: The Joanna Briggs Institute
GRADE-CERQual: Grading of Recommendations Assessment, Development and Evaluation-Confidence in the Evidence from Reviews of Qualitative research
