# Supplementary material for: An exploration of how developers use qualitative evidence: content analysis and critical appraisal of guidelines
Source: BMC Med Res Methodol. 2020 Jun 17;20:160. doi: 10.1186/s12874-020-01041-8 (PMC7302150; doi:10.1186/s12874-020-01041-8)
Supplement: Supplementary file 1 — Additional file 1. The process of data extraction. [file 12874_2020_1041_MOESM1_ESM.doc]

**Additional file 1** The process of data extraction

| Items | Methods of accessing the information |
| --- | --- |
| How was the involvement of experts proficient in qualitative research in the guideline development group determined? | Guideline development group members were identified and their research fields ascertained to determine whether these were related to qualitative research. |
| How was the use of qualitative research to identify clinical questions determined | The introduction on how clinical questions were generated was searched in the guideline text or relevant supplementary file to ascertain the methods for question generations for example, were relevant persons interviewed to ascertain their values and preferences, were clinical questions generated based on the results of existing qualitative research articles. |
| How was retrieval of qualitative evidence determined? | The search strategy in the guideline text or relevant supplementary files was checked to determine whether authors had used relevant search terms related to qualitative research such as “qualitative research or qualitative interview or ethnography ”. In addition, we traced the reference of the evidence supporting the recommendation to determine whether the reference is based on qualitative research by reading the full text version, where this is present, qualitative evidence may be said to have been retrieved. |
| How was the use of qualitative evidence to support recommendations determined? | The reference of the evidence supporting recommendations was traced to determine whether it is based on qualitative research by reading the full text version. |
| How was the application of qualitative evidence to the consideration of facilitators and barriers to implementation of recommendations' determined | The introductory section on how to consider facilitators and barriers of recommendations' implementation was accessed and checked in the guideline text or relevant supplementary files. In addition, we have checked each recommendation and evidence summary to determine whether qualitative evidence was applied to consider the facilitators and barriers to the recommendations' implementation. |
| How was the theoretical basis of the qualitative research used, identified? | The reference of the qualitative evidence supporting the recommendation was traced and the full text read to determine which qualitative research theoretical basis is used. In addition, where the guideline development group had used qualitative research or relevant articles to identify clinical questions, we checked the introduction on how to obtain clinical questions in the guideline text or relevant supplementary files, and relevant qualitative research articles in order to determine which qualitative research theory basis was used. |
| How was the quality assessment tool used for qualitative research identified? | The methodology introduction on how to assess the quality of the qualitative research articles used was accessed in the guideline text or relevant supplementary files on quality assessment results. |
| How was the quality level of primary qualitative research studies used to formulate recommendations identified? | The quality level indicators of primary qualitative research studies presented after each evidence summary were identified and checked by looking in the guideline text or relevant supplementary files such as summary of quality evaluation results to identify the quality level of primary qualitative research studies |
| How was the quality level of qualitative evidence synthesis used to formulate recommendations identified? | The quality level indicators of qualitative evidence synthesis presented after each evidence summary were identified and checked by looking in the guideline text or relevant supplementary files such as summary of quality evaluation results to identify the level of quality of the qualitative evidence synthesis |
| How was the level of qualitative research in the grade criteria of evidence and recommendations determined? | The methodology introduction on grading criteria of evidence and recommendation was accessed and checked by looking in the guideline text, relevant supplementary files or guideline development handbook to determine which level of qualitative research is described in the grade criteria of evidence and recommendations |
| How was the grade of those recommendations which were supported only by qualitative evidence determined? | The quality level indicators of grade of recommendations were accessed in the guideline text and checked for those recommendations supported only by qualitative evidence. |
| How was the grade of those recommendations supported by qualitative and quantitative evidence determined? | The quality level indicators of grade of recommendations were accessed in the guideline text and checked for those recommendations supported by both qualitative and quantitative evidence |
